# An Eco-Friendly Hydrophobic Deep Eutectic Solvent-Based Dispersive Liquid–Liquid Microextraction for the Determination of Neonicotinoid Insecticide Residues in Water, Soil and Egg Yolk Samples

**DOI:** 10.3390/molecules25122785

**Published:** 2020-06-16

**Authors:** Rawikan Kachangoon, Jitlada Vichapong, Yanawath Santaladchaiyakit, Rodjana Burakham, Supalax Srijaranai

**Affiliations:** 1 Creative Chemistry and Innovation Research Unit, Department of Chemistry and Center of Excellent for Innovation in Chemistry, Faculty of Science, Mahasarakham University, Maha Sarakham 44150, Thailand; wittisit@gmail.com; 2 Department of Chemistry, Faculty of Engineering, Rajamangala University of Technology Isan, Khon Kaen Campus, Khon Kaen 40000, Thailand; sanyanawa@gmail.com; 3 Materials Chemistry Research Center, Department of Chemistry and Center of Excellent for Innovation in Chemistry, Faculty of Science, Khon Kaen University, Khon Kaen 40002, Thailand; rodjbu@kku.ac.th (R.B.); supalax@kku.ac.th (S.S.)

**Keywords:** hydrophobic deep eutectic solvent, two disperser solvents, extraction, neonicotinoid insecticides, HPLC

## Abstract

A green, simple and sensitive hydrophobic DES-based dispersive liquid–liquid microextraction coupled with high-performance liquid chromatography (HPLC) was developed for the analysis of neonicotinoid insecticide residues in various samples. A hydrophobic deep eutectic solvent (DES) was synthesized using decanoic acid as a hydrogen bond donor and tetrabutylammonium bromide (TBABr) as a hydrogen bond-acceptor. DESs were synthesized and characterized by Fourier transform-infrared (FTIR) spectroscopy. Two disperser solvents were substituted with surfactants and acetonitrile, which could afford more effective emulsification and make the extraction relatively greener. The hydrophobic DES extraction phase occurred 10 min after centrifugation, being easy to be collected for analysis. Several parameters were investigated and optimized. Under the optimum condition, the calibration curve of this method was linear in the range of 0.003–1.0-µg·mL^−1^, with a correlation coefficient (R^2^) higher than 0.99 and a good repeatability, with the relative standard deviations (RSDs) were less than 5.00%. The limits of detection were in the range of 0.001–0.003 µg·mL^−1^; the limits of quantitation were in the range of 0.003–0.009 µg·mL·mL^−1^. Finally, the presented method was implemented to determine the neonicotinoid insecticide residues in water, soil, egg yolk samples and acceptable recoveries were obtained.

## 1. Introduction

In chemistry, the choice of the right solvent is essential, since it constitutes around 80% of the total volume of chemicals used in a process [1]. Solvents present numerous environmental, health and safety challenges including human and ecotoxicity issues, process safety hazards and waste management issues [2]. Following the principles of green chemistry, most of the organic solvents do not fulfil the requirements for their use in green technology, since they have an inherent toxicity and a high volatility [1]. The challenge of these volatile and hazardous organic solvents may reside in health, the environment pollution produced and the unacceptable solvent residues in the extraction [3]. In addition based on an environmental perspective, these solvents pose significant challenges in green chemistry. Therefore, the development of a new alternative green extraction technique and the search for alternative solvents are imperative [4]. Until recently, the research has been focused on ionic liquids (ILs) as an extraction medium due to their unique properties, such as density, viscosity, hydrophilicity or hydrophobicity and solubility, which could be adjusted by the selection of an appropriate cation and anion [5,6]. Beside mention advantages for ILs, due to high price, tedious synthesis routes, need for further purification of the synthesized ILs and toxicity of some cases lead to limitation of their extensive applications [7]. An alternative to classical ILs are deep eutectic solvents (DESs), which have similar properties, but their synthesis is simpler and less expensive, and they are more biodegradable and often less toxic than ILs [8]. DESs are a mixture of a halide salt or another hydrogen bond acceptor (HBA) and one or two hydrogen bond donors (HBDs) [9]. DESs are eutectic mixtures for which the eutectic point temperature should be lower to that of an ideal liquid mixture [10] and some superior properties such as low vapor pressure, high thermal stability, biodegradation and biocompatibility [11]. Most of the early DESs were water soluble, which limited their application with water containing samples, and development of hydrophobic DES has attracted much attention in order to extend the range of applications [12]. Most DESs which have been introduced up to now are water-miscible [13]. The hydrophilicity of DESs limits their applicability in the extraction of hydrophobic compounds [14]. Recently, the preparation and application of hydrophobic DESs was reported, such as using decanoic acid and various quaternary ammonium salt [15], menthol-based hydrophobic low viscosity solvents [16], indium extraction from hydrochloric and oxalic acids using hydrophobic DESs and low-transition-temperature mixtures [15], thus greatly enlarging the possibilities of DESs.

Therefore, sample preparation is an important preliminary step before analysis [14]. Dispersive liquid–liquid microextraction (DLLME) has been considered one of the most promising analytical methods and has attracted much attention because of its simply, efficiency, low cost and high enrichment factors (EFs) with a broad variety of acceptor and donor phases [17]. DLLME is based on the formation of fine droplets of an extraction solvent in an aqueous sample solution. When a water-immiscible organic dispersive solvent is rapidly injected into the aqueous sample solution, the analytes in sample solution are extracted into the fine droplets and which are further separated by centrifugation [18]. However, Rezaee and coworkers recently reported a DLLME as a powerful technique, but the main drawback of such methodology is the use of highly toxic and polluting extraction chlorinated solvents [19]. The development of “green extraction agents”, which meet the requirements for an extraction agent in conventional LLME operations, but also substantially reduce the volatility and toxicity of the extraction agents (thus reducing the dangers to operators) has, therefore, attracted increasing attention [12].

The determination of neonicotinoid insecticide residues is usually challenging due to their low concentration and the presence of various interfering substance in real samples. Neonicotinoids are a relatively new class of insecticides chemically related to nicotine. In less than 20 years, neonicotinoids have become the most widely used class of insecticides. Their presence now accounts for at least one quarter of the world’s insecticide market [20]. They are nicotinic acetylcholine receptor agonists interfering with the transmission of neural messages in insects more efficiently than mammals and vertebrates [21]. These compounds are most commonly used in rice, maize, sunflower, rape, potato, sugar beet, vegetables and fruit crops [22]. Many counties have formulated strict limits about the neonicotinoids in various matrices. The European Union (EU) legislation has established standards/regulations for the maximum residue limits (MRLs) for neonicotinoid insecticides in different agricultural products. The MRLs for neonicotinoid insecticides in fruits, vegetables, and cereals are between 0.1–1.0 mg·kg^−1^ [23]. The accumulation of insecticides in agricultural products is of great concern because plants act as intermediates in the transport of contaminates from soil, water and air to human and fauna [24]. Therefore, sensitive method is required for monitoring and determination of trace levels of neonicotinoid insecticide residues in various samples.

Neonicotinoids are unsuitable for the direct analysis by gas chromatography (GC) due to their low volatility and high polarity [14]. The use of high-performance liquid chromatography (HPLC) coupled with various detection system including ultraviolet (UV) [25], diode array (DAD) [26], fluorescence (FRD) [27] and mass spectrometry (MS) [28] is the preferred choice for neonicotinoid insecticides analysis. However, the MS detector provided more selectivity and sensitivity than UV detector for monitoring of target analytes in complex matrices, it is a very expensive and complex instrument [29].

The aim of this research was to introduce a hydrophobic deep eutectic solvent (DES) based on decanoic acid mixed with tetrabutylammonium bromide (TBABr) as extraction solvent for dispersive liquid–liquid microextraction (DLLME) method combined with high-performance liquid chromatography (HPLC). The main factors influencing extraction were investigated and optimized, such as salt addition, types of disperser solvent and volume of hydrophobic DES. The optimum conditions for extraction and determination of neonicotinoid insecticide residues in surface water, human urine and soil samples.

## 2. Results and Discussion

### 2.1. Characterization of Hydrophobic DES (TBABr with Decanoic Acid)

**Figure 1:**
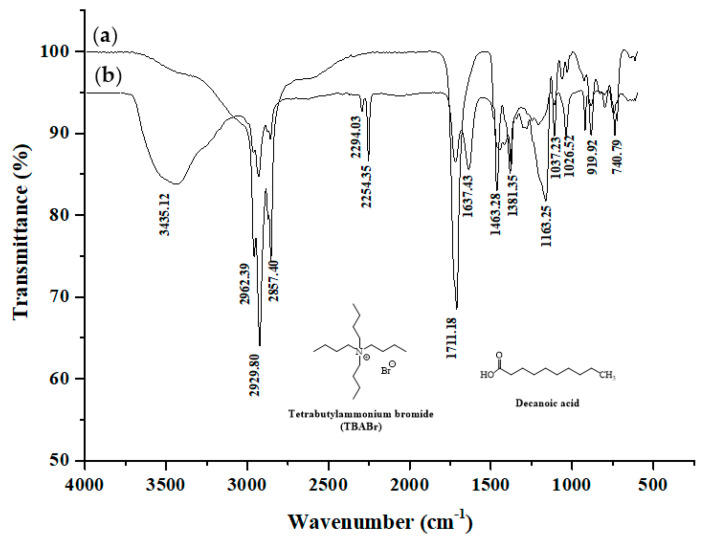
The Fourier transform infrared (FTIR) spectra of (**a**) hydrophobic deep eutectic solvents (DESs) and DES with acetonitrile (ACN) and (**b**) sodium dodecyl sulfate (SDS).

In addition, in order to elucidate the interactions between the two components resulting in the formation of hydrophobic DESs, FTIR spectra were recorded. A comparison of FTIR spectra of pure components [30,31] and the DESs formed is shown in Figure 1. The synthesis of hydrophobic deep eutectic solvents is accomplished by the formation of hydrogen bonds between HBA and HBD. The location of the bonds depends on the structure of the reactants. An inspection of FTIR spectra of all the investigated hydrophobic deep eutectic solvents also reveals a O-H band of decanoic acid at 3435.12 cm^−1^, C=O band at 1716.73 cm^−1^, C-O band at 1037.23, methylene group (-CH_2_) band at 2857.40 cm^−1^ and methyl group (-CH_3_) band at 2929.80 cm^−1^ [32]. FTIR spectra of tetrabutylammonium bromide (TBABr) reveals a methylene group (-CH_2_) and methyl group (-CH_3_) band at wavenumbers: 2854.37 cm^−1^, 2923.23 cm^−1^ and 2955.80 cm^−1^, respectively. Appearance of the C–H asymmetric stretching vibration band at lowered wave number with low intensity suggested that the vibration of the methyl groups is somehow restricted due to inclusion. The methylene scissoring vibration band disappeared in hydrophobic DES and methylene twisting vibration band was shifted to 1381.35 cm^−1^. This is because of the trapping of TBAB hydrophobic tail into analytes. Interestingly, in hydrophobic DES the C–N stretching vibration appeared at 1026.52 cm^−1^ coinciding to that of the pure TBABr [30]. This fact clearly signified that the positively charged nitrogenous head group of TBABr just protrude outside the analytes. In addition. the spectra of DESs formed from TBABr and decanoic acid reveal a characteristic shift of the bands corresponding to stretching vibrations of the carbonyl group towards higher wavenumber: from 1711.18 cm^−1^ to 1716.73 cm^−1^, which indicates the formation of new hydrogen bonds in the vicinity of the COOH group. Thus, the shift of the O–H vibrations suggests the existence of hydrogen bonding between TBABr and decanoic acid when the hydrophobic DES is formed.

### 2.2. Optimization of Hydrophobic Deep Eutectic Solvent Based on Dispersive Liquid–Liquid Microextraction Procedure

In order to obtain the high extraction efficiency of the proposed method, several parameters were investigated and optimized, including molar ratio and volume of hydrophobic DES, type and volume of disperser solvents, type and concentration of surfactant, salts addition and extraction time. To identify of optimal extraction conditions, the peak area of the analytes was applied to evaluate extraction efficiency under the various conditions. In this experiment, various parameters were studied by a one parameter at a time while the other factors were kept constant. The optimization was carried out on the aqueous solution (10.00 mL) containing 0.50 µg·mL^−1^ of each analyte. All experiments were performed in triplicate and average value was used for optimization.

#### 2.2.1. Selection of Hydrophobic DES and Its Volume

The composition of hydrophobic DES has significant influence on its physicochemical properties, which may greatly affect the extraction efficiency of target analytes [12]. Five different mole ratios of hydrophobic DES (1:1, 2:1, 3:1, 4:1, 5:1) based on decanoic acid mixed with TBABr were prepared and their ability to extract the five neonicotinoid insecticides in various samples were investigated (data not shown). The result of ability for extraction using DES (mole ratio 4:1) provided higher extraction efficiency in term of peak area, but it is not stable (solidified at room temperature). Thus, in this method hydrophobic DES (mole ratio 3:1) was used for further experiment.

**Figure 2:**
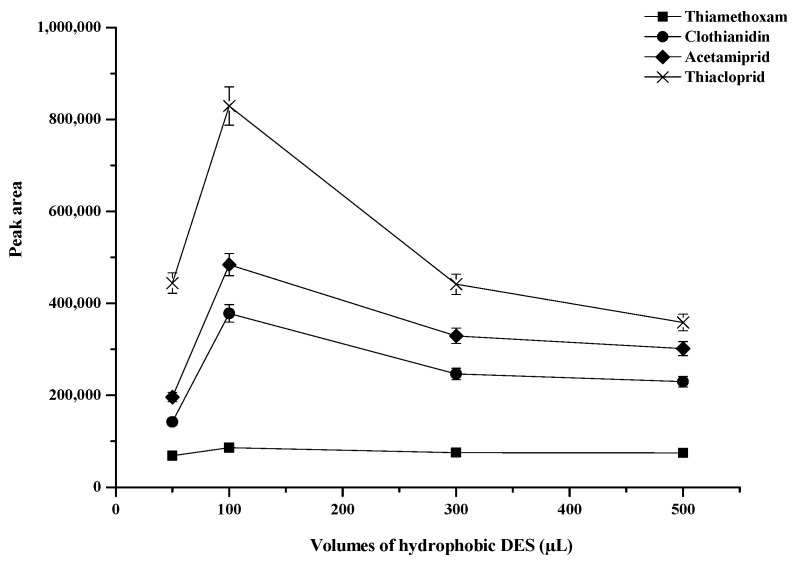
Effect of the volume of hydrophobic DES. Conditions: 400 µL of ACN; 10 mmol·L^−1^ of SDS; vortex time 30 s; centrifugation time at 5000 rpm for 10 min (sample volume 10 mL, 0.5 µg·mL^−1^ of each neonicotinoid insecticide).

The volume of hydrophobic DES (mole ratio 3:1) has influence on the extraction efficiency. Different volumes of hydrophobic DES (mole ratio 3:1) (50, 100, 300, 500, 700, 1000 μL) were investigated, the result is shown in Figure 2. It was found that an enhancement of extraction efficiency for all neonicotinoid insecticides in term of peak area when 100 μL hydrophobic DES (mole ratio 3:1) was added. At the volume of hydrophobic DES (mole ratio 3:1) higher 100 μL (300–1000 μL), peak of clothianidin and imidacloprid was overlap and the large volumes of hydrophobic DES (mole ratio 3:1) did not increase efficiencies because dilution effect. Therefore, the volume of 100 μL was selected as extraction solvent.

#### 2.2.2. Effect of Type and Volume of Disperser Solvents

**Figure 3:**
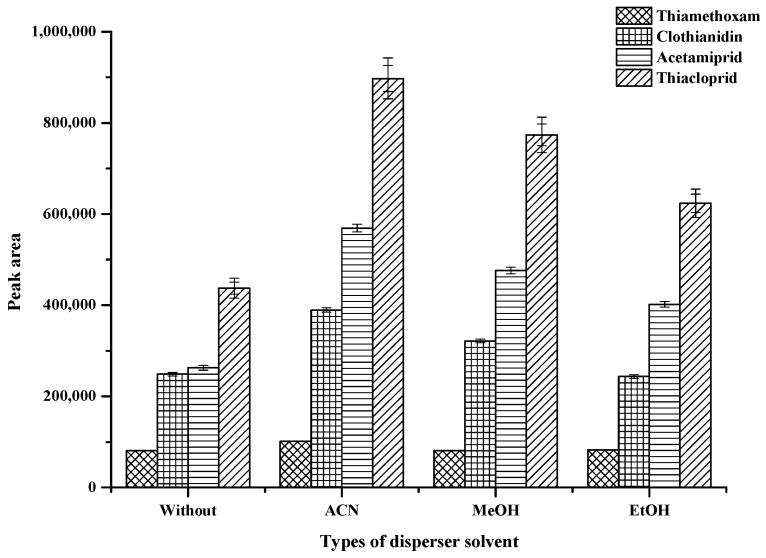
Effect of types of disperser solvent. Conditions: 100 µL of 0.1-mol L^−1^ SDS; vortex time 60 s; 100 µL of hydrophobic DES (mole ratio 3:1); centrifugation time at 5000 rpm for 10 min (sample volume 10 mL, 0.5 µg mL^−1^ of each neonicotinoid insecticide).

In the proposed method, experiment for choosing the disperser solvents were performed using 400 μL of acetonitrile (ACN), methanol (MeOH) and ethanol (EtOH) and the results were compared with that obtained from the procedure without disperser solvent. The result is shown in Figure 3. Among of these ACN was found to provide the highest extraction efficiency due to cooperate effect of good compatibility of ACN with aqueous solutions and the low distributive ratio of analytes in mixture of ACN and water [33]. Thus, ACN was chosen as the disperser solvent for further experiments.

The volume of ACN (50, 100, 200, 400, 600, 800 and 1000 μL) in the presence of constant volume of 50 μL SDS and 100 μL hydrophobic DES were tested (data not shown). It was found that extraction efficiency increased up to 400 μL of the disperser solvent and then decreased due to their high solubility in the aqueous phase [31] in the presence of high volume of ACN and at 1000 μL of ACN, no phase separation was obtained. As a result, 400 μL of ACN was selected.

#### 2.2.3. Effect of Type and Concentration of Surfactant

The selection of the surfactant also greatly influenced the developed microextraction procedure. The structure of surfactants affects its physical and chemical properties, which may affect the extraction efficiency of key factors [34]. In this method, different types of surfactant including sodium dodecyl sulfate (SDS), cetyltrimethyl ammonium bromide (CTAB), Triton X-100 (TX-100) and Triton X-114 (TX-114) at 100 μL were investigated and the results were compared with that obtained from the procedure without surfactant (data not shown). It was found that the addition of SDS provided higher extraction efficiency in term of peak area of studied neonicotinoid insecticides. A surfactant aggregate orientates its hydrocarbon tails towards the center to create a nonpolar core. Isolated hydrophobic substances, which is presented in the aqueous solution, is favorably partitioned in the hydrophobic core of micelles [33]. Thus, SDS was selected.

The effect of concentration of SDS on the extraction efficiency were studied in the range of 3–100 mmol·L^−1^ (data not shown). When increasing the concentration of SDS from 3 to 10 mmol·L^−1^, the extraction efficiency increased and constant at 30 mmol·L^−1^. Moreover, the concentration of SDS from 50 to 100 mmol·L^−1^, more turbid solution and no phase separation was obtained. Therefore, the concentration of 10 mmol·L^−1^ SDS was used.

#### 2.2.4. Effect of Salts Addition

**Figure 4:**
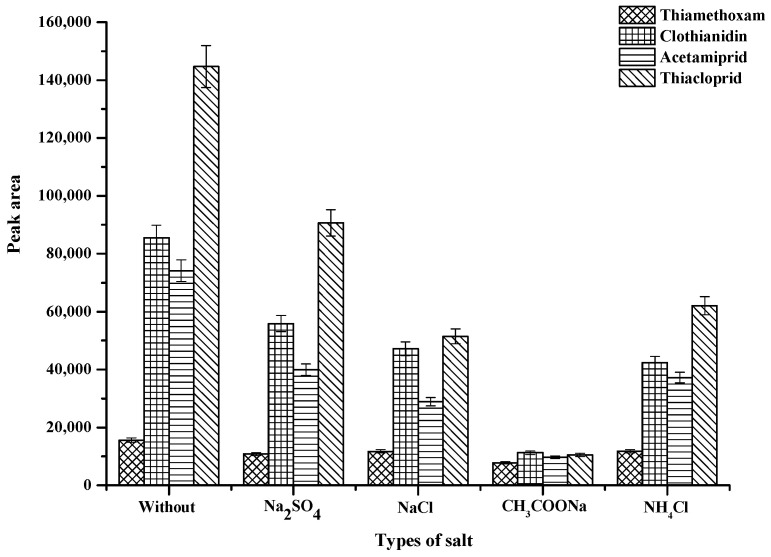
Effect of types of salt. Conditions: 400 µL of ACN; 100 µL of 0.1-mol·L^−1^ SDS; vortex time 60 s; 100 µL of hydrophobic DES (mole ratio 3:1); centrifugation time at 5000 rpm for 10 min (sample volume 10 mL, 0.5 µg·mL^−1^ of each neonicotinoid insecticide).

The addition of salt to aqueous solutions mainly cases decrease in the solubility of organic solvents in water, the addition of salt has been widely used to improve the extraction recovery of analytes [31]. To study the effect of ionic strength on the proposed method, different electrolyte salts such as NaCl, Na_2_SO_4_, CH_3_COONa and NH_4_Cl at 0.3 g were investigated. The results were compared with that obtained from the procedure without salt addition. The experiment result is shown in Figure 4, indicated that the extraction efficiency decreased with adding the different electrolyte salts in term of peak area of the studied neonicotinoid insecticides. Thus, no salt was added in subsequent experimental.

#### 2.2.5. Effect of Vortex and Centrifugation Time

In order to increase the vortex time speeds up the distribution equilibrium of the target analytes between hydrophobic DES (mole ratio 3:1) and aqueous solution and improve the recovery (data not shown). The vortex time speed was evaluated in the range of 10–150 s compared with that obtained from the process without vortex agitation (data not shown). The extraction efficiencies were improved with extraction time over the range 10–60 s, with a maximum at 30 s. Moreover, the vortex time more than 60 s, no phase separation was occurred. Therefore, the vortex time of 30 s was selected.

To achieve the phase separation, centrifugation time is another important step. The centrifugation speed was fixed at maximum speed of instrument (5000 rpm) and fixed time of 10 min, to ensure complete phase separation in the proposed microextraction method.

### 2.3. Analytical Performance of the Proposed Method

**Figure 5:**
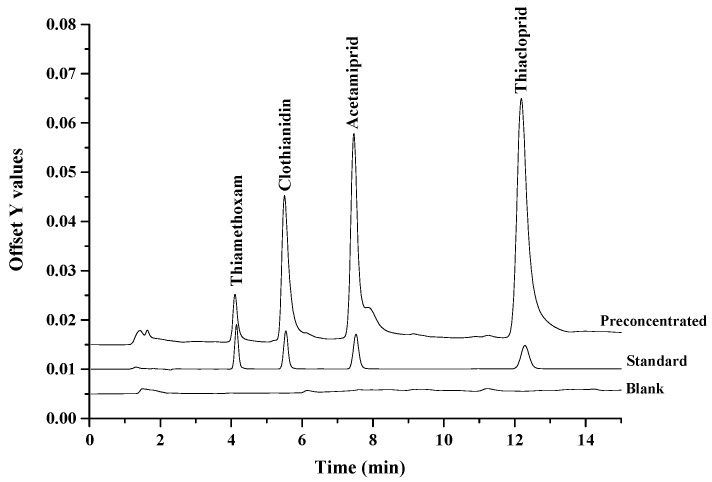
The overlaid chromatograms of the studied neonicotinoid insecticides obtained from blank, direct high-performance liquid chromatography (HPLC) and preconcentrated by the proposed microextraction method (sample volume 10 mL; 0.1 µg·mL^−1^ of each neonicotinoid insecticide).

The analytical performance of developed hydrophobic deep eutectic solvent based on dispersive liquid–liquid microextraction method coupled with HPLC-UV for determination of neonicotinoid insecticide residues in various samples was evaluated in term of linear range, limits of detection (LODs), limits of quantitation (LOQs), repeatability, extraction recovery and enrichment factor (EF) of each target analyte. The analytical performances of the proposed method were performed by enriching 10 mL of working standard solution, and the results as summarized in Table 1. The proposed method gives a good linearity in the range of 0.003–1 μg·mL^−1^ with the correlation coefficient (R^2^) greater than 0.99. The sensitivity was characterized by limits of detection as the concentration providing a signal-to-noise of 3 and limits of quantitation as the concentration providing a signal-to-noise of 10, LODs were in the range of 0.001–0.003 μg·mL^−1^ and LOQs were in the range of 0.003–0.009 μg·mL^−1^, respectively. Four neonicotinoid insecticide solutions of 0.01 and 0.10-μg·mL^−1^ were employed to determine the precision as the relative standard deviation. The relative standard deviations of the retention times and peak areas were less than 5.00%. The enrichment factor (EF), defined as the concentration ratio of the analytes in the settled phase (C*_set_*) and in aqueous samples (C_0_), ranged from 10 to 30-fold. Chromatograms of the studied neonicotinoid insecticides obtained from direct HPLC and preconcentrated by the proposed microextraction method are shown in Figure 5.

### 2.4. Analysis of Real Samples

**Figure 6:**
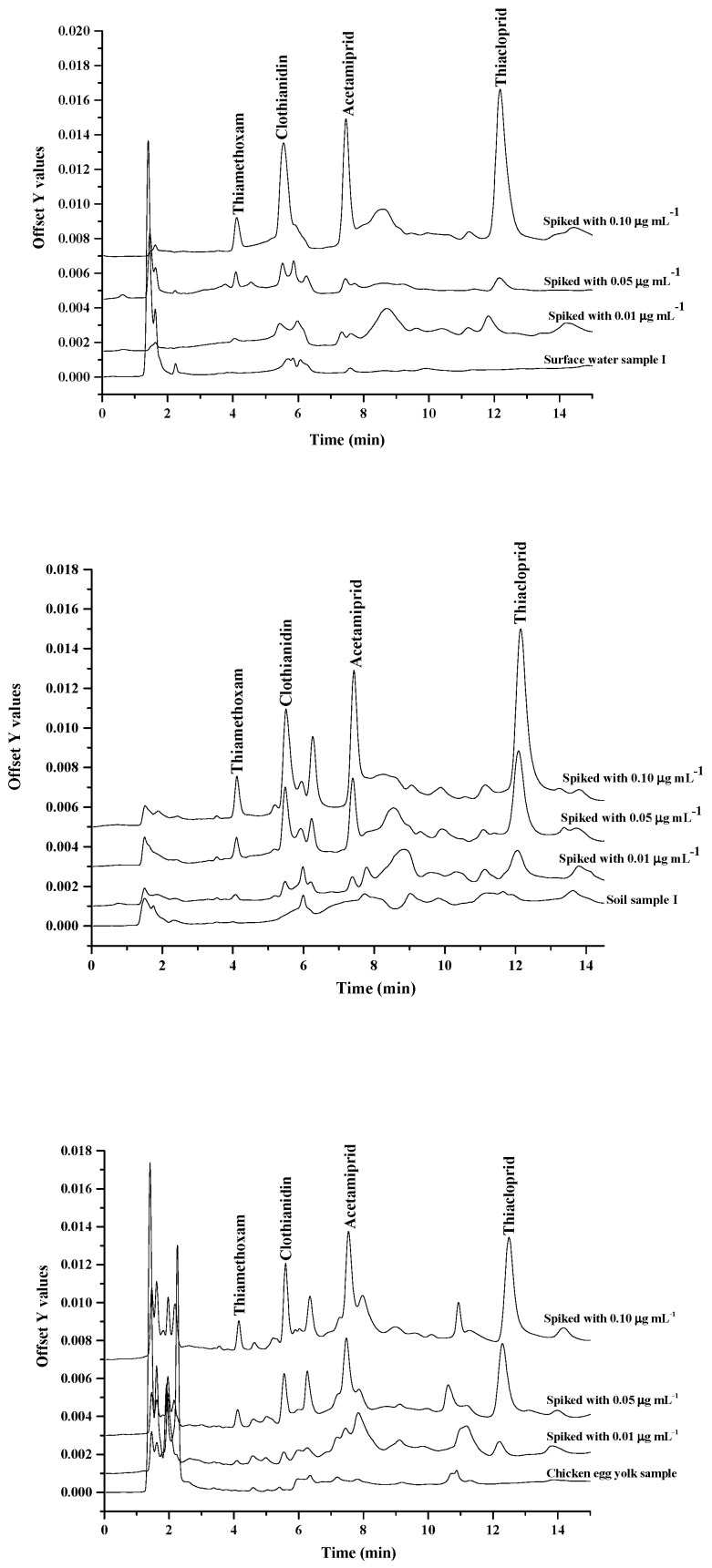
The overlaid chromatograms of sample and spiked samples.

For evaluating of the applicability and recovery of the proposed method, three types of sample including surface water, soil and egg yolk samples were examined. These samples were spiked with the neonicotinoid insecticides at the different concentration of 0.01, 0.05 and 0.1 μg·mL^−1^, before extraction and analysis. The overlaid chromatograms of samples and spiked samples are shown in Figure 6. The recoveries of the studied neonicotinoid insecticides were obtained in the range of 60–114% (data not shown). The relative standard deviations (RSDs) were less than 10.0%, at the estimated spiking different concentration levels. The obtained LODs of this method is lower than the maximum residue limit (MRL) established by EU. For the studied samples, no neonicotinoids insecticide residues were detected in the studied samples.

### 2.5. Comparison of the Proposed Hydrophobic Deep Eutectic Solvent Based on Dispersive Liquid–Liquid Microextraction With Other Sample Preparation Methods

Comparisons between the current DLLME–hydrophobic DES method and previous sample pretreatment methods for the preconcentration and determination of neonicotinoid insecticide residues are shown in Table 2. The results showed that the parameters of this method such as linearity, LODs, LOQs and recovery were the same as or greater than those of most of the reported methods. Moreover, the advantages of the DLLME–hydrophobic DES method can be summarized as follows: (i) the extraction solvent (hydrophobic DES) are a new generation of green solvents, which is necessary from environmentally friendly and economical attitudes, (ii) simple preparation procedures, the components (HBD, neutral compounds such as carboxylic acid, alcohol, sugars and salts) can be easily mixed to obtain target eutectic mixture, (iii) the proposed method is miniaturized, making it possible to reduce dramatically the amounts of samples, reagents and solvents consumed and wastes generated. The present method achieves low LODs which are below the MRLs established by EU for neonicotinoid insecticide residues in environmental and animal products.

## 3. Experimental

### 3.1. Chemicals and Reagents

The analytical standards of neonicotinoid including thiamethoxam, clothianidin and acetamiprid were obtained from Dr. Ehrenstorfer GmbH (Augsburg, Germany); thiacloprid was purchased from Fluka (Munich, Germany). The stock solution of each neonicotinoid insecticides (1000-μg·mL^−1^) was prepared by dissolving an appropriate amount in methanol. A series of working solutions of each standard was prepared by diluting the stock standard solution with water. Tetrabutylammonium Bromide (TBABr) was purchased from ACROS Organics (Belarus, Belgium). Decanoic acid (C_10_H_20_O_2_) was purchased from Sigma-Aldrich (Steinheim, Germany). Sodium dodecyl sulfate (SDS; C_12_H_25_NaSO_4_), Triton X-100 and Triton X-114 were purchased from Merck (Darmstadt, Germany). Cetyltrimethyl ammonium bromide (CTAB; C_19_H_42_BrN) was purchased from Calbiochem (Darmstadt, Germany). Methanol (CH_3_OH) and acetonitrile (CH_3_CN) of HPLC grade were obtained from Merck (Darmstadt, Germany). Sodium sulfate anhydrous (anhydrous Na_2_SO_4_), sodium chloride (NaCl), sodium carbonate (Na_2_CO_3_) and ammonium chloride (NH_4_Cl) were obtained from Ajax FineChem (Auckland, New Zealand). Sodium acetate (CH_3_COONa) was obtained from Carlo Erba (Val de Reuil, France). All chemicals were of at least analytical reagent grade and deionized water (Millipore Waters, Milford, MA, USA) with the resistivity of 18.2 MΩ·cm was used throughout the experiments.

### 3.2. Chromatographic Conditions

A Waters 1525 Binary HPLC pump (Waters, Milford, MA, USA) coupled with Waters 2489 UV-Visible detector was used for chromatographic detection. A Purosphere^®^ STAR RP-18 endcapped (4.6 × 150 mm; 5 µm) column (Merck, Darmstadt, Germany) was used as an analytical column carried out at room temperature. Empower 3 software was used for the system control and data processing. A Rheodyne injector was used. The injection volume was 20 µL. The mobile phase was consisted of acetonitrile and water (25:75, *v/v*) was delivered at a flow rate of 1.0 ML·min^−1^. The detection of studied neonicotinoid insecticides was set at a wavelength of 254 nm.

### 3.3. Synthetic of Hydrophobic Deep Eutectic Solvent

The hydrophobic DESs were prepared using different molar ratio (1:1, 2:1, 3:1, 4:1 and 5:1) between decanoic acid and tetrabutylammonium bromide. The mixtures were heated in water bath at 80 °C until transparent clear liquids were obtained. After cooling, the hydrophobic DESs were stored at room temperature.

### 3.4. Hydrophobic Deep Eutectic Solvent-Based Dispersive Liquid–Liquid Microextraction

**Figure 7:**
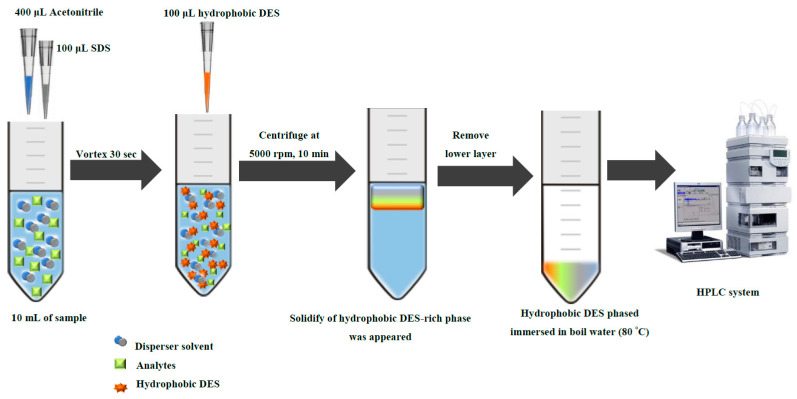
Schematic for a hydrophobic deep eutectic solvent-based dispersive liquid–liquid microextraction.

A schematic diagram of microextraction procedure is shown in Figure 7. Exactly 10.00 mL of standard or sample solution was transferred to centrifuge tube. Then, 400 μL of acetonitrile and 100 μL of 0.1-mol·L^−1^ SDS were added. The mixture was vortex for 30 s. After this, we added 100 μL of hydrophobic DES into the centrifuge tube. During this period, the aggregated hydrophobic DES droplets gently decomposed into minute droplets and were homogeneously dispersion in the aqueous sample. Then, a turbid solution was obtained and centrifuged at 5000 rpm for 10 min. At this time, the hydrophobic DES-rich phase was occurred. Hydrophobic DES phased was kept and immersed in boil water (80 °C) for melting the solid phase. Finally, 20 μL of the hydrophobic DES phase was injected into the HPLC system for further analysis.

### 3.5. Sample Preparation

#### 3.5.1. Surface Water

Three surface water samples were collected from the different natural located near agricultural in Maha Sarakham province, Northeast Thailand. These samples were filtered through a Whatman No. 1 filter paper and kept at 4 °C before the proposed extraction method.

#### 3.5.2. Soil

Three soil samples were collected from the different natural located near agricultural in Maha Sarakham province, Northeast Thailand. These samples taken from the surface (0–10 cm depth). Soils were air-dried, ground and sifted through a 2-mm sieve. After this, samples were extracted using the method proposed by Meghesan-Breja et al. [40] and Arnnok et al. [41]. In detail, the accurately weighed 20 g soil sample was mixed with 20 mL of water, then 20 mL of acidified acetonitrile (1% acetic acid) was added. The samples were mechanically shaken at 200 rpm for 10 min before adding 24 g of anhydrous sodium sulfate and 6 g of anhydrous sodium acetate, after which the mixture was shaken by hand for a few minutes. The supernatant was subsequently transferred to a 50-mL centrifuge tube. A 10-mL aliquot of the upper layer was taken and mixed with water up to 125 mL and kept at 4 °C before applying to the proposed extraction method.

#### 3.5.3. Egg Yolk

Chicken, duck and quail eggs were purchased from local markets in Maha Sarakham province. It was necessary to separate the yolk from the white, since in the analysis of eggs collected from animals treated with anthelmintics it is known that the concentrations are greater in the yolk [42,43]. Fortification of the samples, when necessary, was performed directly on the yolk and a period of about 12 h was allowed to elapse before continuing with any of the extraction processes in order to improve the interaction between the analytes and the matrix compounds [44]. In detail, an aliquot of 10.00 g of egg yolk were mixed well with 0.2 g of anhydrous Na_2_SO_4_. 1% (*v/v*) Acetic acid in acetonitrile (2.00 mL) was added and shaken vigorously by hand for 1 min, and the homogenized eggs were centrifuged at 3500 rpm for 5 min for complete fat and protein precipitation. The supernatants were collected by micro syringe. The solutions were diluted with deionized water to 10.00 mL, 100 μL of acetic acid was added, and the solutions were centrifuged to ensure complete precipitation of fat and proteins [42]. The samples were spiked with the neonicotinoid insecticides at different concentrations (0.01, 0.05 and 0.10-μg·mL^−1^) before fat and protein precipitation. Thus, the obtained clear solutions were subjected to dispersive liquid–liquid microextraction using hydrophobic DES as extraction solvent, and the hydrophobic DES rich phase was then analyzed by HPLC.

## 4. Conclusions

In this work, an efficient hydrophobic DES was synthesized from decanoic acid and tetrabutylammonium bromide (TBABr). Based on the use of hydrophobic DES, a green, fast and inexpensive DLLME technique was proposed for the preconcentration, extraction and separation of neonicotinoid insecticide residues coupled with HPLC-UV. Low limits of detection (LODs) were 0.001–0.003-μg·mL^−1^ for all target analytes which below the acceptable maximum residue limits (MRLs) established by EU for neonicotinoid insecticide residues. The proposed method is simple and efficient in the analysis of neonicotinoids in surface water, soil and egg yolk samples. Although the application of DESs in environmental analysis will grow in the near future, there is still a need for further investigations using DESs as extractive agents. For example, new hydrophobic DESs with different polarities should be synthesized to improve their application to various samples.

## Figures and Tables

**Table 1:** Analytical performance of the proposed method for four different neonicotinoid insecticides.

Analyte	Linear Range (µg·mL^−1^)	Linear Equation	r^2^	LOD (µg mL^−1^)	LOQ (µg mL^−1^)	RSD (%)				EF
						t_R_ (*n* = 5 × 3)		Peak Area (*n* = 5 × 3)		
						Intra Day	Inter Day	Intra Day	Inter Day	
Thiamethoxam	0.003–1	y = 203,253x + 366.71	0.9996	0.001	0.003	0.10	0.20	1.37	4.13	10
Clothianidin	0.003–1	y = (1 × 10^6^)x + 691.91	0.9991	0.001	0.003	0.19	0.29	1.67	3.42	10
Acetamiprid	0.009–1	y = 875,385x − 2215.8	0.9989	0.003	0.009	0.18	0.21	1.07	3.88	30
Thiacloprid	0.009–1	y = (2 × 10^6^)x − 5337	0.9994	0.003	0.009	0.32	0.46	1.63	4.57	30

**Table 2:** Comparisons of the proposed method with other methods for the quantitation of neonicotinoid insecticides.

Method *.	Linear Range	Extraction Solvents	Solvent Usage	Extraction Time	LOD	% Recovery	Ref.
DLLME-LC-MS/MS	1.5–100.0 μg·kg^−1^	acetonitrile/dichloromethane	2.5 mL	20 min	0.5–1.0 μg·kg^−1^	74.3–113.9	[28]
PSE-LC-MS/MS	-	MeOH	5 mL	20 min	0.8–1.5 μg·kg^−1^	83.2–101.9	[35]
LLE-LC-ESI-MS	2.0–1000 μg·kg^−1^	n-hexane/isopropanol (8:2%*v/v*)	15 mL	18 min	0.6–2.3 μg·kg^−1^	85–105	[36]
QuEChERS-LC-MS/MS	1.56–400 μg·L^−1^	1% acetic acid in acetonitrile	10 mL	50 min	1.0–3.0 μg·kg^−1^	72–104.8	[37]
Capillary-ESI-MS	4.0–1000 μg·L^−1^	n-hexane/isopropanol (8:2%*v/v*)	15 mL	25 min	2.6–4.7 μg·L^−1^	60–71	[38]
DLLME-HPLC-DAD	1.5–100 μg·L^−1^	acetonitrile/dichloromethane	2.5 mL	17 min	1.5–2.5 μg·L^−1^	73.1–118.3	[39]
Hydrophobic DES-DLLME-HPLC-UV	3–1000 μg·L^−1^	hydrophobic DES	600 μL	10.3 min	1–3 μg·L^−1^	60–114	This work

* PSE-LC-MS/MS—Pressurized solvent extraction and liquid chromatography-tandem mass spectrometry; LLE-LC-ESI-MS—Liquid–liquid extraction coupled with electrospray ionization mass spectrometry; DLLME-LC-MS/MS—Dispersive liquid–liquid microextraction coupled with liquid chromatography tandem mass spectrometry; QuEChERS-LC-MS/MS—Quick; easy; cheap; effective; rugged and safe method coupled with liquid chromatography tandem mass spectrometry; Capillary-ESI-MS—Capillary electrophoresis-mass spectrometry; DLLME-HPLC-DAD—Dispersive liquid–liquid microextraction-high-performance liquid chromatography coupled with diode array detector; DLLME-HPLC-UV—Dispersive liquid–liquid microextraction-high-performance liquid chromatography coupled with ultraviolet detection.
